# Inverted U-Shaped Relationship between Physical Activity and Academic Achievement among Chinese Adolescents: On the Mediating Role of Physical and Mental Health

**DOI:** 10.3390/ijerph19084678

**Published:** 2022-04-13

**Authors:** Tianjiao Wang, Congbin Guo

**Affiliations:** Institute of Economics of Education, Peking University, Beijing 100871, China; st20453d@gse.pku.edu.cn

**Keywords:** physical activity, physical health, mental health, adolescents

## Abstract

We aimed to clarify the relationship between Chinese adolescents’ physical activity and academic achievement, the mediating role of physical and mental health, and provide a scientific reference for the integration of sports and education. Based on baseline data from the China Education Panel Survey, we conducted a nationwide study of 12,960 adolescents. We used nonlinear models, mediation effect analysis, and other measurement methods. Three significant findings emerged: (1) adolescent physical activity duration and academic achievement showed an inverted U-shaped relationship. Moderate physical activity improves academic achievement. The inverted U-shaped inflection point is about 3.549 h/week; (2) physical activity among the male group has a stronger effect on promoting academic achievement, while the positive effect of physical activity among females is not significant. The difference in effectiveness between urban and rural youth groups is relatively small, but the inflection point of rural youth groups is higher, and (3) the level of physical and mental health effectively mediates the inverted U-shaped influence of youth physical activity on academic achievement. We recommend that governments and schools should implement physical activity appropriately and suggest the feasibility of using physical activity to narrow the growth gap between urban and rural youth.

## 1. Introduction

In past research, the effect of physical activity on adolescents’ academic achievement and cognitive development has been extensively discussed. In a review study, Barbosa et al. concluded that existing studies found that physical activity behavior does not harm the academic achievement of adolescents, and may be beneficial [1]. Regarding the direct influence mechanism of physical activity on the academic achievement and cognitive ability of adolescents, there is no definite conclusion yet [2,3], but the development of neurophysiology in recent years has provided us with some basic evidence. Neurophysiological research has suggested that physical activity can improve attention, information processing, storage and retrieval, and enhance the positive effect by increasing oxygen saturation and glucose delivery [4]. It may also induce neural growth and modification in synaptic transmission, resulting in changes in thinking and decision-making, particularly in the prefrontal cortex, to promote students’ academic achievement and cognitive development [5]. In terms of the law of influence, the experimental study conducted by Fleck [6] found that the influence of physical activity on academic achievement may be nonlinear. In the study, the cognitive ability test was performed by subjecting subjects to a 60% VO_2_ max intensity cycling exercise; results showed that subjects experienced a significant cognitive decline after exercising for more than 120 min. In recent years, Syvaoja also found an inverted U-shaped relationship between the intensity of students’ physical activity and their academic achievement in a Finnish survey [7]. The study used the frequency of physical activity as a measurement standard and found that 5–6 times a week is the optimal frequency for adolescents. In contrast to the above findings that physical activity can effectively improve adolescents’ cognitive ability and academic achievement, some studies have found that physical activity is not significantly correlated with personal academic achievement or cognitive ability [8,9,10,11]. These differing research conclusions may be caused by insufficient control of confounding factors. Follow-up research should consider the control variables added to the model more comprehensively [1].

In addition to the direct influence mechanism, studies have also focused on the effect-quantity relationship between physical activity and adolescents’ academic achievement, and the moderating effects of related variables. The standardized quantitative research on such problems can be traced back to 1996 when Shephard conducted an experimental study on 546 adolescents in Canada. He found that adding one hour of physical activity a day can significantly improve the academic achievement of adolescents, and the effect on girls is more obvious than that on boys [12]. However, Garst found no significant gender difference in the impact of physical activity on academic achievement, and this finding may be related to a series of factors, such as differences in countries, research methods, and social development [13]. Using a large-scale questionnaire survey of American high school students, Guest et al. found that physical activity can improve the academic achievement of young people. They also noticed that the social environment and different physical intensities of physical activity both have regulatory effects on physical activity results [14]. In recent years, Chinese scholars have gradually begun to focus on the impact of physical activity on adolescents’ academic achievement. Zhang et al. found a significant positive correlation between physical activity and adolescents’ academic achievement in their research [15]. While drawing similar conclusions, Dong et al. found that non-cognitive abilities represented by environmental adaptability played an important mediating role in the impact of physical activity on academic achievement [16]. However, the existing studies lack in-depth research on the regulatory effect of gender and environment, as well as the nonlinear relationship between physical activity duration and academic achievement.

As research on the direct relationship between adolescent physical activity and academic achievement is relatively mature, an increasing number of scholars have begun to pay attention to the possible intermediary factors, among which the intermediary role of physical health and mental health is particularly valued. This is mainly because the physical and mental health of young people is of great significance to their personal development and human capital accumulation [17]. The goal of integrating sports and education proposed by the Chinese government in 2020 is to promote the healthy growth of young people [18]. In terms of physical health, many recent studies have demonstrated the positive impact of physical activity on the health of children and adolescents [19,20,21,22]. Grieco et al. verified the important role of physical activity in improving adolescents’ body mass index (BMI) levels and found that children’s academic achievement correlated with their physical fitness levels [23], meaning that physical activity and improving their fitness level may improve their academic achievement [24]. In terms of mental health, Chinese researchers have found that physical activity can improve the mental health of Chinese adolescents [25,26,27]. Several international studies have confirmed that physical activity has a positive effect on anxiety [28,29], depression [30,31,32], psychological stress [33,34,35], and mental disorders [36]. The important role of adolescents’ mental health in academic achievement has also been verified in previous studies [36,37]. Based on the discovery of the link between youth physical activity, physical and mental health, and academic achievement in existing research, there are few studies using quantitative analysis methods to explore the intermediary impact of adolescents’ overall physical health and mental health on the effect of physical activity on academic achievement.

This study will use a large-scale adolescent survey’s data from China to estimate the nonlinear relationship between physical activity and academic achievement in adolescents, using quantitative analysis to try to find the optimal range of physical activity duration in adolescents. Based on this, the study will also explore the differences in the relationship between physical activity and academic achievement across gender and urban-rural contexts to gain a clearer understanding of this issue. Finally, this study employs a cutting-edge mediating effect estimation method to explore the mediating role played by adolescent physical and mental health in the nonlinear relationship between the effects of physical activity on academic achievement.

## 2. Materials and Methods

### 2.1. Population and Procedure 

The data used in this study were obtained from the 2013–2014 baseline survey of the China Education Panel Survey (CEPS), which was designed and implemented by the National Survey Research Center (NSRC) of Renmin University of China. As one of China’s representative education panel survey projects, this open data set supports many empirical studies in the field of education, and is highly reliable. The data obtained in this survey have also been widely used in research on Chinese education issues [38,39]. The survey covered the entire country. First, 28 districts were selected from 2870 across the country as the primary sampling frame, and four schools were selected from each district by adopting probability proportional to scale sampling methods as the secondary sampling frame. Finally, two classes were randomly selected from each school as a third-level sampling frame. If a school has only one or two classes, all samples were taken. The final survey selected 19,487 adolescents from 438 classes in 112 schools. After deleting observations with missing key information, the final sample size of the study was 12,960.

### 2.2. Variables

The dependent variable was adolescents’ academic performance scores. The data were obtained from the mid-term exam scores of young people in the fall semester of 2013–2014 obtained by CEPS from the surveyed schools, including the scores of the three main subjects: Chinese, mathematics, and English. The mid-term exam was organized by the school where the students were surveyed. Since China provides a unified syllabus for the teaching of junior high school, the content of the teaching and assessment of each junior high school was basically the same. The test scores were provided to the investigators by the person in charge of teaching at the school or the main teacher of the class, which prevented the deviation of the scores reported by young people. The scores for each subject were standardized. Standardized scores were calculated separately by school and grade, and adjusted to the mean = 70 and standard deviation = 10. By adding the scores of the three courses, the academic scores of the main subjects of the teenagers were obtained. As adolescents’ performance came from schools’ independent propositions and paper marking, this study adds school-fixed effects to the regression analysis to make the adolescents’ performance comparable. The core explanatory variable was the number of hours the adolescents participated in physical activity each week. The questionnaire contained two items: “Last Monday to Friday, how many hours and minutes did you participate in physical activity every day on average?” As far as the connotation range of physical activity is concerned, the types of physical activity were not explicitly restricted or exemplified in the questionnaire, in order to avoid the restriction of physical activity items that would lead adolescents to underestimate their physical activity hours in the process of filling out the questionnaire. However, considering that the survey respondents were all adolescent students in compulsory education at school, and that China provides students with syllabus-based physical education courses at the compulsory education level, including running, basketball, soccer, calisthenics, etc., the surveyed samples had a high degree of consistency in their perceptions of physical activity. The method of collecting exercise time in the form of self-report has been widely used in existing studies, and the reliability and validity of this method have also been widely recognized [15,16].

The core variable was derived by adding these two self-reported items as the total number of physical activity hours of adolescents in a week. Among all adolescents, 16.5 h of physical activity per week is in the 95th quantile. We used this as the singular value division standard and deleted the observations that reported more than 16.5 h of exercise per week. The intermediary variable was the physical and mental health of adolescents, including physical and mental health. Considering that it is difficult to show the overall health level of adolescents by simply using indicators such as BMI and the degree of myopia, this study drew on Guan’s research, and measured adolescents’ health status self-assessment by adopting a five-point scale “How is your overall health now (1 = very bad to 5 = very good)?” [40]. In terms of mental health, we drew on the measurement methods of existing Chinese research [41]. This study used the five questions on mental health in the questionnaire, “Did you have the following feelings in the past 7 seven days: depressed/depressed/unhappy/life meaningless/sad? (1 = never to 5 = always)” to set to *x*_1_ − *x*_5_ after reverse scoring, and then the mental health level score *E_i_* of teenager *i* was formula 1 by the principal component analysis method (Cronbach’s α reliability coefficient = 0.861, *p* < 0.01). The higher the score, the higher the mental health level.

In terms of control variables, the control variables at the individual level of adolescents included: “gender” (1 = boys, 0 = girls); “grade” (1 = 9th grade, 0 = 7th grade); “household type” (1 = rural household, 0 = non-rural household); “only child” (1 = yes, 0 = no); “cognitive ability score” (measured by the standardized score of cognitive ability test conducted by the CEPS project); “live on campus” (1 = Yes, 0 = No); and “Hours of extracurricular study per week” (the sum of the time reported by teenagers to write to parents after school, the time for homework assignments in tutoring class, and the time to attend extracurricular tutoring class).

The control variables at the family level included adolescents’ families’ “Social Economic Index (SEI).” We performed principal component analysis on the family economic income reported by adolescents, the types of parents’ occupations, and the educational level of their parents. The SEI of the adolescent’s family was calculated using the factor score coefficient. The larger the value, the higher the socioeconomic status of the adolescents’ families. “Parental education expectations” (4 = Expect to complete higher education, 3 = Expect to complete high school education, 2 = Expect to complete compulsory education, 1 = It doesn’t matter); “Strictness of parental discipline” (according to eight aspects of parents’ performance in homework, examinations/school performance/study every day/when to go home every day/who to make friends with/to dress up/the time to surf the Internet/the time to watch TV from questionnaire test, we used principal component analysis factor score coefficients to calculate the parental discipline strictness index. The higher the index, the higher the parental strictness).

To control for school-level factor variables, this study controlled for school-fixed effects by adding school dummy variables to the econometric model. It made adolescents’ academic achievement extracted from the school comparable, and effectively controlled school-level factors such as school management mode, schoolwork pressure, and hardware facilities that may confuse the relationship between physical activity and adolescents’ academic achievement.

### 2.3. Data Processing and Analysis

We first conducted descriptive statistics and one-way ANOVA tests on the variables involved in the study to show the distribution of sample characteristics and preliminarily explore the correlation between physical activity duration and teenagers’ academic achievement and other personal characteristics. Then, we built a nonlinear regression model to analyze the relationship between the duration of physical activity and academic achievement. Finally, to analyze the mechanism of academic performance during adolescents’ physical exercise in depth, we adopted a new nonlinear mediation effect test method to examine the mediating effect of physical and mental health levels. 

The data were analyzed using SPSS22.0 (IBM, Chicago, IL, USA) and stata13.1 statistical software (StataCorp, College Station, TX, USA). First, based on the nonlinear relationship model summarized by Haans et al. (see Equation (1)), the nonlinear relationship between the length of adolescent physical activity and academic achievement was verified. The three conditions are as follows: (1) the coefficient *β*_2_ in the model is significantly negative; (2) assuming that *X_L_* is the minimum value of the independent variable value range and *X_H_* is the maximum value of the independent variable value range, then the slope at *X_H_* (*β*_1_ + 2*β*_2_*X*_H_) is positive, the slope at *X*_H_ (*β*_1_ + 2*β*_2_*X*_H_) is negative; (3) the inflection point (−*β*_1_/2*β*_2_) of the model is within the value range of variable *X*. If the three conditions above are met, it can be considered that there is an inverted U-shaped nonlinear relationship between *X* and *Y.*

*Y* = *β*_0_ + *β*_1_*X* + *β*_2_*X*^2^ (1)

Based on this, we constructed an inverted U-shaped nonlinear education production function model between physical activity duration and academic achievement (2):

GRADE*_i_* = α_0_ + α_1_HOUR*_i_* + α_2_HOUR*_i_*^2^ + α_3_CONTROL*_i_* + α_4_SCHOOL*_i_* + *ε_i_* (2)

where GRADE*_i_* represents the academic achievement of youth *i*, HOUR*_i_* is the hours of physical activity, HOUR*_i_*^2^ is the square term of the hours of physical activity, CONTROL*_i_* is a vector set composed of control variables at the adolescent individual and family level, and SCHOOL*_i_* represents the school fixed effect.

Although the “three-step” method is a classic method to test the mediation effect, it has been questioned in recent years [42,43]. Especially for the nonlinear relationship that this study focused on, Hayes et al. believe that this method may lead to the introduction of bias in the estimation of the inverted U-shaped relationship in the mediation path [44]. Therefore, this study adopted the nonlinear mediation test method proposed by Hayes et al., and used the instantaneous indirect effect (denoted as θ) to test the mediation effect. The θ value is the product of the partial derivative of the intermediate variable HEALTH with respect to the explanatory variable HOUR, and the product of the explained variable GRADE with the partial derivative of HEALTH (see Equation (3)). According to the value of HOUR, the sample is divided into lower (mean minus one standard deviation), moderate (sample mean), and higher (mean plus one standard deviation) three-level groups. We calculated θ at each level value and obtained the confidence interval using the bootstrap method. If the interval does not include 0, then the mediation effect of the mediation variable is significant. This study assumed that there is an inverted U-shaped nonlinear relationship among the three variables of HOUR, HEALTH, and GRADE, and used the Medcurve macro developed on SPSS by Hayes et al. to conduct the test.

(3) $$\theta =\frac{\partial \mathrm{HEALTH}}{\partial \mathrm{HOUR}}\;\frac{\partial \mathrm{GRADE}}{\partial \mathrm{HEALTH}}$$

## 3. Results

### 3.1. Descriptive Statistics and Differences in Characteristics

Overall, as shown in Table 1, 12,960 adolescents were included in the sample, and the proportion of boys was 50.4%. In the sample, 50% of the adolescents were in the ninth and seventh grades, respectively, 52.7% of the adolescents were from rural households, 45.9% belonged to one-child families, and 31.8% of the adolescents were living on campus. In the sample, the average weekly hours of physical activity for teenagers was about 2.92 h, the average hours of extracurricular learning was about 12.915 h, and the average weekly family labor hours was about 7.644 h.

According to the distribution of physical activity time of samples, in the case of trisection samples, the physical activity times of teenagers at two trisection points were 0 h and 3 h, respectively. Since the Chinese government put forward the initiative of “exercising for half an hour every day” to improve the participation of adolescents in physical exercise, we integrated the sample distribution and practical significance and divided adolescents into three groups, according to not participating in physical activity, 0 h < physical activity duration < 3.5 h, and 3.5 ≤ physical activity duration, and compared the differences in characteristics of adolescents among the groups. As can be seen by an intuitive comparison of the mean differences between the three groups, the total score of academic achievement, level of mental health, and cognitive ability score were different between the groups. Both are the highest in the group of teenagers whose weekly physical activity duration is greater than 0 and less than 3.5 h. The physical fitness level was the highest among the adolescents who exercised more than 3.5 h per week.

### 3.2. The Inverted U-Shaped Relationship between the Duration of Physical Activity and Academic Achievement of Adolescents

Models 1–10 report that our baseline regression of academic achievement and heterogeneity test estimation results included nonlinear model results with square-term variables and complementary linear model regression results. In the linear regression model, physical activity duration and academic achievement showed significant negative relationships in all models. However, in the case of the nonlinear relationship, we cannot simply assume that physical activity duration has negative effects on academic achievement, since the positive effects of physical activity on academic achievement over a certain time range may be obscured by the negative effects produced by excessive physical activity duration.

Model 2 in Table 2 showed that in controlling the personal characteristics of adolescents, the duration of study and family labor, the level of family socioeconomic status, the strictness of parental discipline, and parental education expectations, the regression coefficient of adolescent physical activity duration on academic achievement in the case of the school-fixed effect was significantly positive; additionally, the square term of physical activity duration was significantly negative. The slope at HOUR_L_ was positive, the slope at HOUR_H_ was negative, and the model inflection point was within the value range of the variable HOUR, which satisfied the judgment standard of the inverted U-shaped nonlinear relationship. It can be considered that there is an inverted U-shaped nonlinear relationship between the length of adolescent physical activity and academic achievement, and the inflection point was approximately 3.549 h/week. This means that if the weekly physical activity time was less than 3.549 h, physical activity can improve the academic achievement of young people, but the effect weakens after this threshold is exceeded.

Models 4 and 6 tested the effect of the duration of physical activity and academic achievement on adolescents of different genders, respectively. For the male population sample, the regression coefficient of the duration of adolescent physical activity on academic achievement was significantly positive, and the square term of the physical activity duration was significantly negative. The slope at HOUR_L_ was positive, the slope at HOUR_H_ was negative, and the model inflection point was within the value range of the variable HOUR, which satisfied the judgment standard of the inverted U-shaped nonlinear relationship. The model inflection point was 4.514 h/week. Model 6 showed that the regression coefficient of the duration of physical activity in the female group on academic achievement was not significant at the 5% level. The square term of physical activity duration was significantly negative. As the coefficient of the HOUR variable in the model was not significant, the null hypothesis of α1 = 0 cannot be rejected. After α1 = 0 was substituted into the nonlinear test standard, it was found that model 6 did not satisfy the standard with the slope at HOUR_L_. Furthermore, the regression results for the linear model 5 showed that the physical activity duration coefficient was significantly negative at the 1% level. Therefore, the duration of physical activity in the female group only showed a negative impact on academic achievement. 

Models 8 and 10 used the type of adolescent household as the distinguishing standard and tested the effect of the relationship between the duration of physical activity and academic achievement in urban and rural adolescents, respectively. The regression results of the urban youth group showed that the regression coefficient of physical activity duration on academic achievement was significantly positive, and the square term of physical activity duration was significantly negative, the slope at HOUR_L_ was positive, and the slope at HOUR_H_ was negative. The inflection point of the model was within the value range of the variable HOUR. The judgment standard of the inverted U-shaped nonlinear relationship was met, and the model inflection point was approximately 3.453 h/week. The regression results of the rural youth group in Model 5 showed that the regression coefficient of physical activity duration on academic achievement was significantly positive, and the square term of physical activity duration was significantly negative. The slope at HOUR_L_ was positive, the slope at HOUR_H_ was negative, and the inflection point of the model was within the value range of the variable HOUR. The judgment standard of the inverted U-shaped nonlinear relationship was met, and the model inflection point was approximately 3.964 h/week. The inflection point of the model for the urban youth group was 3.453 h/week, which was slightly lower than 3.964 h/week for the rural youth group.

### 3.3. The Mediating Effect of Adolescents’ Physical and Mental Health

Considering that the traditional “three-step” method of estimating nonlinear mediating effects may produce biased estimates, it is impossible to intuitively compare the mediating effects of different explanatory variable values. This study used the nonlinear intermediary test method proposed by Hayes et al., and used the instantaneous indirect effect θ to perform a more reliable analysis. As the test method used the mean of the explanatory variable plus or minus one standard deviation as the grouping test criterion, the left deviation of the physical activity duration in this study caused the value to be less than 0 after the mean minus one standard deviation. Therefore, the θ value of the lower value group is only for reference and does not provide an actual explanation. Table 3 presents the estimation results. 

As far as the mediating effect of mental health is concerned, the confidence interval of θ between the lower group and the higher group of the explanatory variables did not include 0, indicating that mental health could effectively mediate its positive impact on academic achievement in the low and medium physical activity time groups. In the higher physical activity time group, the mediating effect was not significant. In terms of physical health, the θ confidence intervals in the lower group, the middle group, and the higher group did not include 0, and the θ values were all positive, indicating that the physical fitness level in different physical activity time groups could effectively mediate its positive impact on academic achievement. The θ value of the moderate group was higher than that of the higher group, indicating that the positive mediation effect had a higher effect size in the moderate group.

## 4. Discussion

The purpose of this study was to examine the nonlinear relationship between the duration of adolescent physical activity and academic achievement, and to explore the mediating role of physical and mental health factors. Based on large-scale local education survey data in China, this study found that adolescents’ physical activity and academic achievement had a significant inverted U-shaped relationship, and the inflection point in the inverted U-shaped relationship was 3.549 h/week. This conclusion is consistent with conclusions of previous research on the nonlinear relationship between physical activity and academic achievement [6,7]. Both found the existence of an inverted U-shaped relationship, which expanded the discussion of weekly physical activity duration based on short-term testing and physical activity frequency. In addition, the discussion of the relationship between physical activity and academic achievement in previous studies often lacked control over factors such as SES, gender, and family support of adolescents as a result of data limitations [4]. Based on large-scale micro-survey data, this study controlled for the confounding effect of these important factors on the relationship between physical activity and academic achievement in the model, making the results more robust. 

Compared to the widely recognized role of physical activity in promoting youth PP, more attention should be paid to the negative part of the nonlinear effect [1]. Existing studies rarely mention the negative effects of physical activity. In a few studies that explored the nonlinear relationship between physical activity and academic achievement, Syvaoja et al. proposed that the negative effects may be a result of excessive physical activity restricting adolescents’ extracurricular learning time. Although Van Dijk et al. mentioned the problem of squeezing extracurricular learning time, it has not been verified because of limitations of the data [45]. This study controlled for the variables of adolescents’ extracurricular study time and family labor time in the econometric model. At the same time, control of the school’s fixed effects can weaken the intra-school study time differences caused by differences in inter-school curriculum arrangements and teaching intensity. The results proved that under the condition of controlling the learning time factor, the negative effects brought about by excessive physical activity duration still exist. The use of a time squeeze to explain the negative effects of physical activity requires more rigorous evidence to support it.

In response to this negative effect, this study is more inclined to offer explanations from the perspective of neurophysiological and nonlinear mediation effects. From the perspective of neurophysiology, existing studies mostly assume that the direct influence of physical activity on academic achievement comes from the physiological promotion of increased oxygen saturation and glucose delivery, and induces neural growth and modification in synaptic transmission [4,5]. However, this kind of physiological benefit is limited, and it is attenuated by marginal benefits, which will reduce the benefits of physical activity to teenagers’ cognitive level and academic achievement. Meanwhile, the mediating effect analysis adopted in this study provided evidence for the explanation of this problem. As Fleck found in his research, excessive physical activity will cause athletes to produce symptoms of central fatigue, which will cause an increase in errors during complex tasks and an alteration in perceptual response [6]. In this study, a new nonlinear mediating effect analysis method found that physical activity and adolescents’ physical and mental health had an inverted U-shaped relationship, and that this nonlinear relationship further affected adolescent academic achievement. This showed to a certain extent that excessive physical activity will have an additional burden on the physical and mental health of young people, and that this effect is an important reason for the decline in academic achievement levels caused by excessive physical activity. This conclusion provides a valuable empirical basis for future exploration of the transmission mechanism of the nonlinear relationship between physical activity and academic achievement. In the study of the relationship between adolescents’ physical activity and PP, more attention needs to be paid to the physical and mental health burdens that excessive physical activity may place on adolescents, and urges researchers to use scientific methods to explore reasonable physical activity duration and intensity.

Based on the exploration of the overall effect of the relationship between physical activity and academic achievement, this study also performed a group regression analysis on the difference between gender and the effects of urban and rural areas. The results showed that the relationship between physical activity and academic achievement is quite different between the genders. The higher academic achievement of boys in physical activity is significantly greater than that of girls. This is not in line with the conclusions of the previous studies. Most of the existing studies found that there is no significant gender difference in the positive effect of physical activity on academic achievement [13], or that girls’ academic achievement income in physical activity is significantly higher than that of boys [12,46]. This difference may mainly be due to the measurement of physical activity behavior. Existing studies on the measurement of physical activity behavior were mostly based on experimental standards, or have relatively strict requirements for exercise intensity. However, the measurement of physical activity in this study came from self-reporting by teenagers, and there is no specific limitation on the intensity and category of physical activity. Compared with existing research, the significance of this study’s findings is that it contributes to the understanding of gender differences in the impact of physical activity on academic achievement in Chinese contexts, which may arise from gender differences in Chinese cultural norms regarding the expectations and demands of physical activity and academic achievement for adolescents. On the one hand, it can be a focus for future research; on the other hand, it can enable policymakers to realize the true inefficiency of female physical activity, and to enable educational reforms in a targeted manner.

After regressing the samples of urban and rural adolescents, the study found that the difference between the academic achievement income of urban adolescents in physical activity and rural adolescents is very small, and the inflection point of the urban youth group is 3.453 h/week, which was slightly lower than the rural youth group’s 3.964 h/week, indicating that the rural youth have certain advantages in their physical activity income. This conclusion indicates that physical activity may be an effective means to narrow the academic achievement gap between urban and rural youth. Based on the relationship between physical activity duration and cognitive ability in neuroscience, we hypothesize that this difference may be due to the fact that rural Chinese adolescents have relatively greater motor ability and physical fitness [47], which helps rural adolescents to gain from longer periods of physical activity. Follow-up research is required to explore this idea.

This study has several limitations. First, due to the limitation of available data, this study only used cross-sectional data for regression estimation of the relationship between physical activity and academic achievement. As an education panel survey project, we propose using longitudinal panel data to conduct a more rigorous causal inference analysis to analyze the impact of physical activity on academic achievement. Second, this study used the method of single-dimensional self-report to measure adolescents’ physical activity duration and physical and mental health, which may lead to recall and social expectancy biases. Third, this study did not consider the heterogeneity of specific physical activity types and exercise intensity. On the one hand, this part of the heterogeneity may lead to differences in the relationship between physical activity and the development effect of students; on the other hand, preference differences in the type of physical activity and intensity may be the source of the heterogeneity of effect quantity among groups.

## 5. Conclusions

Overall, the weekly physical activity duration of Chinese teenagers was about 2.92 h. The analysis results showed that adolescents’ physical activity duration and academic achievement presented an inverted U-shaped nonlinear relationship, with an overall model inflection point of approximately 3.549 h/week. Adolescents’ physical and mental health factors effectively mediate this inverted U-shaped relationship. In the discussion of the heterogeneity of physical activity income, this study found that boys can obtain significant positive academic achievement income through physical activity, but that this effect was not found in the female group. The difference between the urban and rural youth groups in China was relatively small. Only the inflection point of the model for rural youth groups at 3.964 h/week was slightly larger than the 3.453 h/week for urban teenagers. Finally, we propose to overcome the bias of the subjective reporting of data by using more objective measurements of physical activity duration and intensity. Intervention experiments and longitudinal investigations can also be used to make more robust causal inferences about the relationship between physical activity and academic achievement.

## Figures and Tables

**Table 1:** Sample characteristics and differences in characteristics.

Variable	Full Sample	Not Participating in Physical Activity	0 h < Physical Activity Duration < 3.5 h	3.5 ≤ Physical Activity Duration
	(n = 12,960)	(n = 4329)	(n = 4510)	(n = 4121)
Total academic score	212.973	210.796	216.560	211.336
Mental health level	7.668	7.469	7.804	7.728
Physical fitness	4.075	3.956	4.089	4.185
Physical activity duration (h/w)	2.920	0.000	1.732	7.287
Boys	0.504	0.464	0.471	0.580
Ninth grade	0.500	0.504	0.500	0.494
Rural household	0.527	0.591	0.533	0.452
Only child	0.459	0.393	0.466	0.520
Cognitive ability score	10.332	9.754	10.729	10.506
Live on campus	0.318	0.376	0.322	0.254
Extracurricular study time (h/w)	12.915	9.675	10.717	18.725
Family working hours (h/w)	7.644	7.058	7.017	8.947
SEI	11.560	10.762	11.631	12.319
Parental education expectations	3.721	3.633	3.770	3.761
Strictness of parental discipline	3.453	3.366	3.483	3.512

**Table 2:** Baseline regression of academic achievement and heterogeneity test estimation results.

Variable	Model 1	Model 2	Model 3	Model 4	Model 5	Model 6	Model 7	Model 8	Model 9	Model 10
	Full Sample	Full Sample	Boys	Boys	Girls	Girls	Urban	Urban	Rural Youth	Rural Youth
Physical activity duration	−0.276 ** (0.055)	0.433 ** (0.146)	−0.199 * (0.080)	0.641 ** (0.214)	−0.362 ** (0.075)	0.176 (0.198)	−0.346 ** (0.074)	0.442 * (0.207)	−0.161 * (0.074)	0.436 * (0.209)
Physical activity duration square term		−0.061 ** (0.012)		−0.071 ** (0.017)		−0.047 ** (0.016)		−0.064 ** (0.016)		−0.055 ** (0.018)
Boys	−11.449 ** (0.373)	−11.566 ** (0.373)					−10.667 ** (0.540)	−10.786 ** (0.540)	−12.054 ** (0.517)	−12.154 ** (0.518)
Rural household	0.552 (0.465)	0.508 (0.464)	0.874 (0.704)	0.799 (0.705)	0.106 (0.606)	0.096 (0.605)				
Ninth grade	3.371 ** (0.373)	3.281 ** (0.388)	3.256 ** (0.585)	3.078 ** (0.585)	3.314 ** (0.508)	3.290 ** (0.507)	2.690 ** (0.569)	2.659 ** (0.568)	3.441 ** (0.539)	3.320 ** (0.540)
Only child	1.651 ** (0.458)	1.650 ** (0.458)	2.615 ** (0.679)	2.575 ** (0.678)	0.746 (0.623)	0.776 (0.623)	1.666 * (0.669)	1.650 * (0.668)	1.391 * (0.644)	1.406 * (0.643)
Cognitive ability score	2.551 ** (0.058)	2.536 ** (0.058)	2.798 ** (0.088)	2.773 ** (0.088)	2.295 ** (0.076)	2.288 ** (0.076)	2.529 ** (0.082)	2.520 ** (0.082)	2.540 ** (0.083)	2.522 ** (0.083)
Live on campus	1.844 ** (0.711)	1.851 ** (0.710)	2.865 ** (1.077)	2.852 ** (1.075)	0.078 ** (0.938)	0.102 (0.937)	0.542 (1.295)	0.622 (1.293)	2.357 ** (0.856)	2.339 ** (0.855)
Hours of extracurricular study per week	0.063 ** (0.012)	0.071 ** (0.012)	0.104 ** (0.019)	0.113 ** (0.019)	0.028 (0.016)	0.034 * (0.016)	0.070 ** (0.017)	0.078 ** (0.017)	0.057 ** (0.018)	0.064 ** (0.019)
Hours of family work per week	−0.161 ** (0.026)	−0.171 ** (0.026)	−0.172 ** (0.019)	−0.183 ** (0.040)	−0.170 ** (0.034)	−0.179 ** (0.034)	−0.174 ** (0.042)	−0.186 ** (0.042)	−0.162 ** (0.034)	−0.170 ** (0.034)
SEI	0.149 ** (0.058)	0.139 * (0.058)	0.155 (0.087)	0.140 (0.087)	0.141 (0.075)	0.134 (0.075)	0.318 ** (0.076)	0.309 ** (0.076)	−0.225 * (0.093)	−0.236 * (0.093)
Strictness of parental discipline	−0.404 (0.318)	−0.474 (0.318)	−0.512 (0.487)	−0.628 (0.487)	−0.380 (0.411)	−0.414 (0.411)	−0.619 (0.459)	−0.697 (0.459)	−0.221 (0.443)	−0.280 (0.443)
Expect to complete compulsory education	−18.917 ** (1.896)	−18.873 ** (1.894)	−16.685 ** (2.963)	−16.643 ** (2.491)	−21.208 ** (3.131)	−21.168 ** (3.129)	−20.968 ** (3.140)	−21.051 ** (3.136)	−16.149 ** (2.433)	−16.025 ** (2.432)
Expect to complete high school education	−7.849 ** (1.135)	−7.781 ** (1.134)	−5.655 ** (1.984)	−5.599 ** (1.610)	−10.138 ** (1.600)	−10.054 ** (1.599)	−10.884 ** (1.673)	−10.818 ** (1.670)	−4.448 ** (1.574)	−4.353 ** (1.574)
Expect to complete higher education	10.623 ** (1.060)	10.548 ** (1.059)	12.560 ** (1.893)	12.430 ** (1.508)	8.258 ** (1.492)	8.243 ** (1.491)	8.122 ** (1.493)	8.009 ** (1.491)	13.607 ** (1.512)	13.605 ** (1.511)
School fixed effect	control	control	control	control	control	control	control	control	control	control
Constant term	175.287 **	175.183 **	159.589 **	161.619 **	182.380 **	182.223 **	176.469 **	176.076 **	186.508 **	186.639 **
Observations	12960	12960	6526	6526	6434	6434	6135	6135	6825	6825
adjusted R-square	0.302	0.304	0.267	0.269	0.260	0.261	0.291	0.293	0.323	0.324

Note: (1) * and ** represent significance levels of 5% and 1%, respectively. (2) The standard error of the coefficient is in parentheses.

**Table 3:** Estimation of the effect of physical and mental health as an intermediary factor in the impact of physical activity duration on academic achievement.

Mediating Variable	Group	Explanatory Variable	Transient Mediating Effect	95%CI	
Mental health	Lower group	−0.740 ^#^	0.358	0.020	0.056
	Medium group	2.920	0.186	0.010	0.029
	Higher group	6.580	0.002	−0.002	0.007
Healthy body	Lower group	−0.740 ^#^	0.015	0.002	0.033
	Medium group	2.920	0.010	0.001	0.120
	Higher group	6.580	0.004	0.0004	0.008

^#^: As the overall distribution of the physical activity duration variable is left-biased, it is of no practical significance to take the lower group to be negative, but only for reference.

## Data Availability

The China Education Panel Survey data used to support the findings of this study have been deposited in the http://ceps.ruc.edu.cn/repository (accessed on 10 May 2021).
